# Production and optimization of bioplastic (Polyhydroxybutyrate) from *Bacillus cereus* strain SH-02 using response surface methodology

**DOI:** 10.1186/s12866-022-02593-z

**Published:** 2022-07-22

**Authors:** Shereen M. Hamdy, Amal W. Danial, Sanaa M. F. Gad El-Rab, Ahmed A. M. Shoreit, Abd El-Latif Hesham

**Affiliations:** 1 Pediatric Hospital, Assiut University, Assiut, 71516 Egypt; 2 Botany and Microbiology Department, Faculty of Science, Assiut University, Assiut, 71516 Egypt; 3 Genetics Department, Faculty of Agriculture, Beni-Suef University, Beni-Suef, 62521 Egypt

**Keywords:** Bioplastic, Polymer, PHB production, RSM, Optimization, Bacteria, 16S rRNA gene sequencing

## Abstract

**Background:**

Polyhydroxybutyrate (PHB) is a biopolymer formed by some microbes in response to excess carbon sources or essential nutrient depletion. PHBs are entirely biodegradable into CO_2_ and H_2_O under aerobic and anaerobic conditions. It has several applications in various fields such as medicine, pharmacy, agriculture, and food packaging due to its biocompatibility and nontoxicity nature.

**Result:**

In the present study, PHB-producing bacterium was isolated from the Dirout channel at Assiut Governorate. This isolate was characterized phenotypically and genetically as *Bacillus cereus* SH-02 (OM992297). According to one-way ANOVA test, the maximum PHB content was observed after 72 h of incubation at 35 °C using glucose and peptone as carbon and nitrogen source. Response surface methodology (RSM) was used to study the interactive effects of glucose concentration, peptone concentration, and pH on PHB production. This result proved that all variables have a significant effect on PHB production either independently or in the interaction with each other. The optimized medium conditions with the constraint to maximize PHB content and concentration were 22.315 g/L glucose, and 15.625 g/L peptone at pH 7.048. The maximum PHB content and concentration were 3100.799 mg/L and 28.799% which was close to the actual value (3051 mg/l and 28.7%). The polymer was identified as PHB using FTIR, NMR, and mass spectrometry. FT-IR analysis showed a strong band at 1724 cm^− 1^ which attributed to the ester group’s carbonyl while NMR analysis has different peaks at 169.15, 67.6, 40.77, and 19.75 ppm that were corresponding to carbonyl, methine, methylene, and methyl resonance. Mass spectroscopy exhibited molecular weight for methyl 3- hydroxybutyric acid.

**Conclusion:**

PHB–producing strain was identified as *Bacillus cereus* SH-02 (OM992297). Under optimum conditions from RSM analysis, the maximum PHB content and concentration of this strain can reach (3100.799 mg/L and 28.799%); respectively. FTIR, NMR, and Mass spectrometry were used to confirm the polymer as PHB. Our results demonstrated that optimization using RSM is one of the strategies used for reducing the production cost. RSM can determine the optimal factors to produce the polymer in a better way and in a larger quantity without consuming time.

**Supplementary Information:**

The online version contains supplementary material available at 10.1186/s12866-022-02593-z.

## Background

Plastics are preferred for a range of applications, and the volume of plastic manufactured and used worldwide is increasing, with over 380 million metric tons produced and utilized annually. Almost 80 million metric tons of plastic waste are liberated annually, causing environmental and direct health issues [1]. Severe soil and water pollution occur due to the slow or non-degradable nature of synthetic plastic also, when burning, causes air pollution. Therefore, governments are looking for alternatives to reduce the use of synthetic polymers [2].

The best alternative sources for petrochemical-derived polymers are biopolymers synthesized from biological sources. Polymers derived from microbial sources are the most favorable among the various biological sources due to their easy production and purification [3]. Polyhydroxyalkanoates (PHAs) have gained extensive scientific community interest due to their physical properties similar to synthetic plastics [4]. The PHA-producing organism must degrade it spontaneously during starvation, thus making the polymer more eco-friendly [5]. PHAs are entirely biodegradable in CO_2_ and H_2_O under aerobic and anaerobic conditions. PHA has several applications in various fields such as medicine, pharmacy, agriculture, and food packaging due to its biocompatibility and nontoxicity nature [6].

PHAs are thermoplastic aliphatic polyesters with linear polymer chains which normally accumulated intracellularly by various microorganisms due to excess carbon sources or limitations in microelements (oxygen, nitrogen, and phosphorus). They can act as carbon reserve compounds and energy storage inside the bacterial cell [7]. PHA can be classified into three categories: Short Chain Length (SCL) consisting of 3-hydroxy acids from 3 to 5 carbon atoms, Medium Chain Length (MCL) consisting of 3-hydroxy acids from 6 to 16 carbon atoms and Long Chain Length (LCL) consisting of 3- hydroxy acids more than 16 carbon atoms in a monomer unit [8]. Polyhydroxybutyrate (PHB) is considered the main member of the PHAs family [9].

The cost of PHA production is very high due to the expensive raw material used in the production process. Several strategies have been used to reduce this cost; including utilizing alternative carbon sources (natural products, industrial wastes, and agro-industrial residues) for production through various fermentation processes [10]. Optimizing bioprocesses is one of the main factors that reduce the production costs of all commercial biotechnology products. The classical optimization process always takes one variable at a time, which gives inaccurate results with vast time consuming, and the interactive influence of different variables on production also cannot be solved with this method. Statistical experimental strategies, including factorial design and response surface methodology (RSM), are more dependable than classical experiments [10]. RSM is a mathematical and statistical technique with individual and interactive effects that take into account improvements in the actual optimization process setup, curvature, and troubleshooting of the weak and the problem points [11]. Several design methods have been applied for optimization like central composite design (CCD), Box–Behnken design (BB), Doehlert Matrix (D), Plackett–Burman (PB) design, and full factorial designs. The CCD is one of the most traditional experimental designs among the different RSM classes, and this strategy is particularly helpful in predicting better substrate concentrations with fewer random errors [10]. Several studies have successfully reported optimizing commercial biotechnology products using RSM, such as optimizing bacteriocin production and increasing its activity [11], optimizing conditions for maximum bacterial biofilm removal using α-amylase produced by *B. subtilis* [12], and optimizing laccase production from *Pleurotus* strain sp. [13].

Therefore, this study aims to optimize the conditions for PHB production by a new *Bacillus* isolate. The factors that affect the PHB production were screened using central composite design (CCD) which develops a mathematical model by finding the combination of significant factors for the design of the experiment. The chemical composition of the extracted PHB was confirmed by ^1^H NMR, ^13^C NMR (Nuclear Magnetic Resonance), and Mass Spectrometric (MS) analysis. Functional groups and surface morphological studies of PHB were analyzed by Fourier Transform Infrared (FTIR) spectroscopic.

## Result

### Identification of the isolate

The PHB-producing organism (DC2) showed morphological, physiological, and biochemical characteristics of the genus *Bacillus* (Table 1). The isolated bacterium was Gram-positive, aerobic, motile and rod-shaped forming endospores. Colonies on nutrient agar have regular margins with pale yellow colonies.

**Table 1:** Morphological and biochemical characterization of PHB-producing organism *Bacillus cereus* SH-02, (+) positive response and (−) negative response to the test

Test	Observation
Gram stain	+
Motility	+
Spore formation	+
Catalase test	+
Oxidase test	–
Urease test	+
Indole test	–
Casein hydrolysis	+
Gelatin hydrolysis	+
Citrate utilization	+
H_2_S production	–
Glucose fermentation	+
Sucrose fermentation	+
Mannitol fermentation	–
Maltose fermentation	+
Fructose fermentation	+
Starch hydrolysis	+
Deoxidization of nitrate	–

**Fig. 1:**
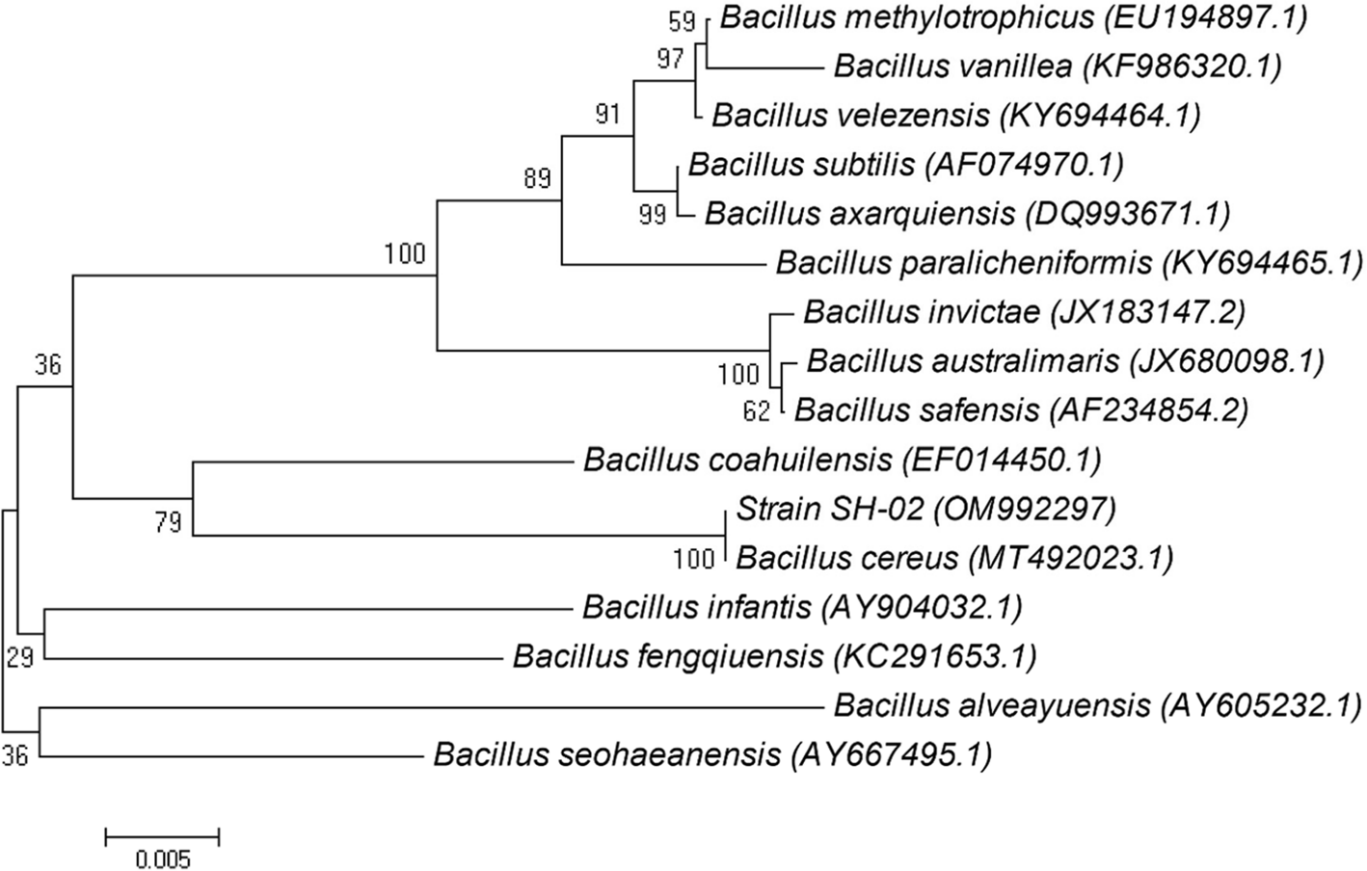
The phylogenetic tree based on the patterns and the genetic relationship of *Bacillus cereus* SH-02 (OM992297)

PHB-production isolate (DC2) was identified according to the 16S rRNA gene. The alignment results showed that the 16S rRNA sequences of the selected strain were highly homologous with % similarities to *Bacillus cereus* (Fig. 1).

### Effect of incubation time on PHB production

**Fig. 2:**
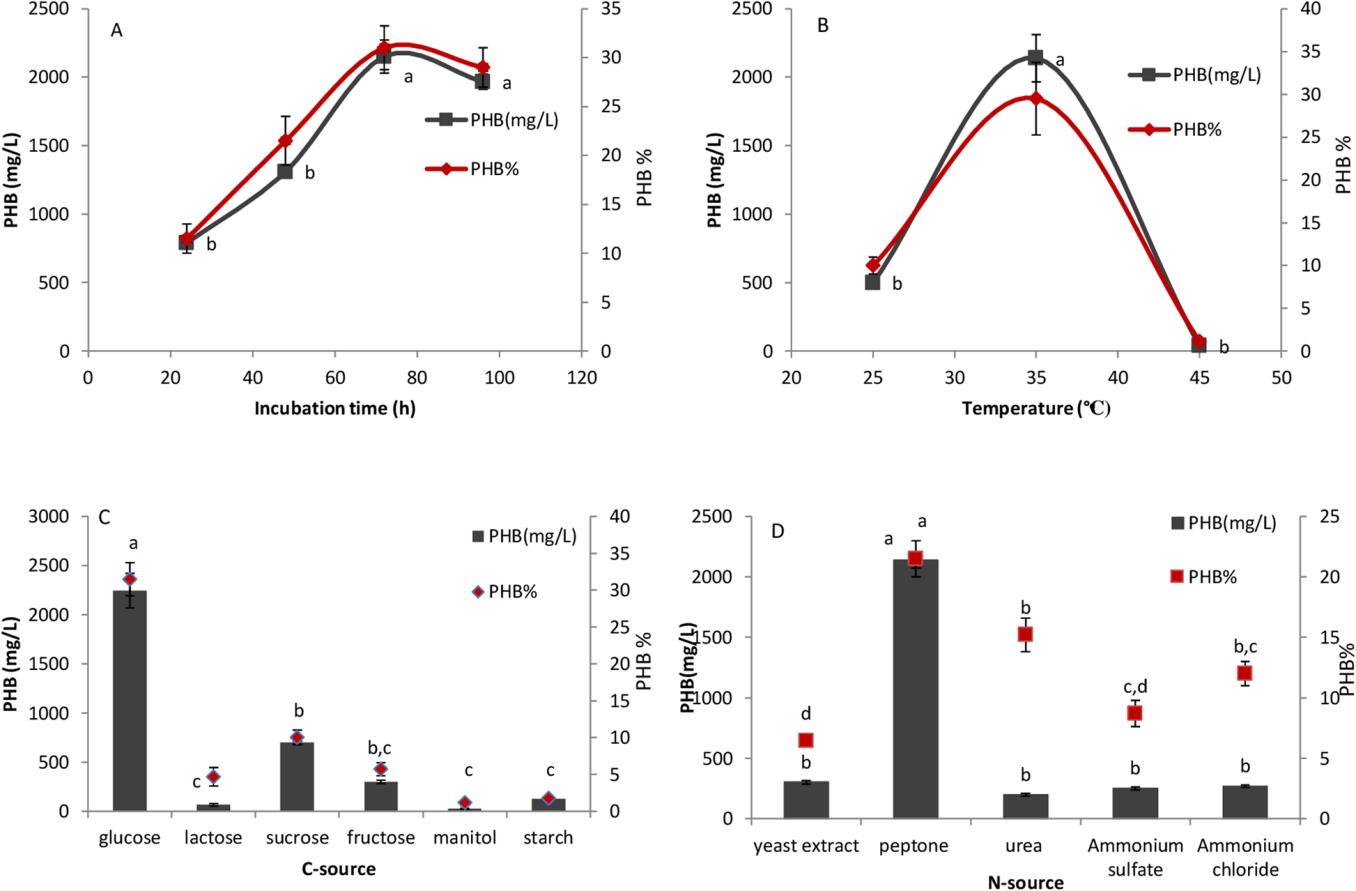
Effect of incubation time (**A**), temperature (**B**), different c-source (**C**) and different nitrogen source (**D**) on polyhydroxy butyrate (PHB) production by *Bacillus cereus* SH-02 (OM992297). The ANOVA test was carried out by using SPSS 21 comparisons among means ± SE standard error (*n* = 3), different letters show significance at *p* = 0.05 level based on Duncan’s multiple range test

The PHB content gradually increased from 789.3 ± 30 mg/L in the initial 24 h of incubation to 2150 ± 120 mg/L at 72 h (Fig. 2A). After that, production decreased because bacteria used PHB as a nutrient source, causing unsuitable conditions for nitrogen and carbon sources in the medium.

### Effect of incubation temperature on PHB production

This study revealed that the most PHB content and concentration (2138.5 ± 174 mg/L and 29.5 ± 4.5%) from *Bacillus cereus* SH-02 (OM992297) were noted at 35 °C than other temperatures (Fig. 2B). That is because the activity of the enzyme responsible for PHB synthesis could be increased under mesophilic temperature than the extreme temperature which could diminish enzymes and protein function [2].

### Screening of different carbon sources for maximum PHB production

Among the different carbon sources screened, glucose was the most suitable source for PHB production (2245 ± 174 mg/L) by *Bacillus cereus* SH-02 (OM992297) (Fig. 2C).

### Screening of different nitrogen sources for maximum PHB production

Supplementation of nitrogen sources in the production media influenced PHB production (Fig. 2D). Among the nitrogen sources screened, peptone was found to be the most suitable nitrogen source for the maximum PHB content and concentration (2137 ± 62.5 mg/L, 21.5 ± 1.5%; respectively) by *Bacillus cereus* SH-02 (OM992297). That may be due to the low nitrogen content of peptone, which increased the C:N ratio, supporting a higher PHB production.

### Statistical optimization of PHB production from *Bacillus cereus* SH-02 (OM992297) strain using response surface methodology (RSM)

PHB-production by *Bacillus cereus* SH-02 (OM992297) was optimized using (Design Expert ver. 12 software, Stat-Ease Inc., Minneapolis, USA). RSM is an effective tool useful for the optimization of media components and other critical variables responsible for the production of biomolecules [14]. Central composite design (CCD) suggested a total of 20 experimental trials with 6 replicates of the central point. CCD model was implemented to optimize PHB production by independent variables such as glucose concentration (A), peptone concentration (B), and pH (C) at five different levels −α (− 1.68), low (− 1), medium (0), high (+ 1) and + α(+ 1.68). Response 1 (PHB content mg/L) and response 2 (PHB concentration) were attained based on the results. Values predicted and measured Analysis of variance (ANOVA) was used to determine whether polynomial expression could statistically predict responses. The regression equation is graphically represented by these counterplots. They are based on the function of the concentration of two major components while keeping the other factors constant. The graphical representation of the regression equation is commonly the 2D contour and 3D response surface plots. The contours of the surface plots can be used to infer the interaction between the variables under investigation [15].

The ANOVA for response 1 (PHB content) quadratic model was tabulated in (Table 2). The following is the second-order polynomial equation for the response 1 of PHB content (mg/L):

$$\mathrm{Response}\ 1:\mathrm{PHB}\ \mathrm{content}\ \left(\mathrm{Y}\right)=+2949.47+459.94\ast \mathrm{A}+408.82\ast \mathrm{B}-72.05\ast \mathrm{C}+140.50\ast \mathrm{A}\ast \mathrm{B}-104.00\ast \mathrm{A}\ast \mathrm{C}+236.00\ast \mathrm{B}\ast \mathrm{C}-747.14\ast {\mathrm{A}}^2-566.17\ast {\mathrm{B}}^2-749.66\ast {\mathrm{C}}^2$$

**Table 2:** ANOVA for Quadratic Model (Response 1:PHB content)

Source	Sum of Squares	Df	Mean Square	F-value	***p***-value
**Model**	2.334E+ 07	9	2.594E+ 06	188.15	< 0.0001^a^
**A-glucose concentration**	2.889E+ 06	1	2.889E+ 06	209.57	< 0.0001^a^
**B-peptone concentration**	2.282E+ 06	1	2.282E+ 06	165.57	< 0.0001^a^
**C-pH**	70,898.58	1	70,898.58	5.14	0.0467^c^
**AB**	1.579E+ 05	1	1.579E+ 05	11.46	0.0069^b^
**AC**	86,528.00	1	86,528.00	6.28	0.0312^c^
**BC**	4.456E+ 05	1	4.456E+ 05	32.32	0.0002^a^
**A**^**2**^	8.046E+ 06	1	8.046E+ 06	583.63	< 0.0001^a^
**B**^**2**^	4.619E+ 06	1	4.619E+ 06	335.10	< 0.0001^a^
**C**^**2**^	8.099E+ 06	1	8.099E+ 06	587.50	< 0.0001^a^
**Residual**	1.379E+ 05	10	13,785.55		
**Lack of Fit**	1.128E+ 05	5	22,553.60	4.49	0.0623 NS
**Pure Error**	25,087.50	5	5017.50		
**Cor Total**	2.348E+ 07	19			
**Std. Dev.**	117.41				
**Mean**	1540.75				
**C.V. %**	7.62				
**PRESS**	9.804E+ 05				
**R**^**2**^	0.9941				
**Adjusted R**^**2**^	0.9888				
**Predicted R**^**2**^	0.9583				
**Adeq Precision**	36.0784				

^a^, Highly significant; ^b^, Significant; ^c^, less significant; *NS* Not significant

**Fig. 3:**
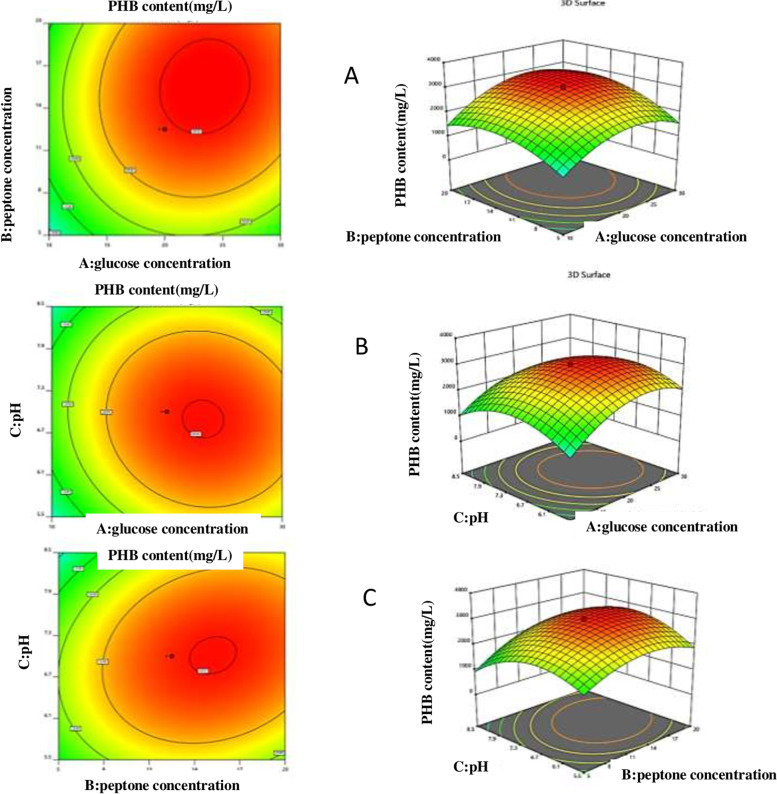
Three-dimensional interactions between of independent factors (**A**) glucose concentration and peptone concentration at standard pH, (**B**) glucose concentration and pH level at standard peptone concentration, (**C**) peptone concentration and pH level at standard glucose concentration

The model F-value of 188.15 shows a significant influence of different variables on PHB content either independently or in interaction with each other (Table 2). In this case, A, B, C, A^2^, B^2^ and C^2^ were significant model terms. The interactive effect of variables BC (peptone concentration and pH) and AB (glucose concentration and peptone concentration) had the most impact on PHB content (Fig. 3A and C) as compared to AC (glucose concentration and pH) (Fig. 3B). The 4.49 “Lack of Fit” F-value indicates that it is small in comparison to the pure error. Non-significant lack of fit is acceptable and the model is suitable. The total determination coefficient R^2^ was 0.9941, indicating that the model suited the experimental data reasonably well (Table 2). This also suggests that 99% of response variance can be properly explained and that 1% of the variability occurs during the experiments [16]. With a coefficient of variation of 7.62%, the corrected determination coefficient value of 0.9888 proved that the model was highly significant. The Adjusted R^2^ value of 0.9888 is reasonably close to the Predicted R^2^ value of 0.9583. The signal-to-noise ratio is measured using “Adeq Precision.” A ratio of more than 4 is ideal. The signal-to-noise ratio of 36.0784 suggests that this model can navigate the design space.

**Fig. 4:**
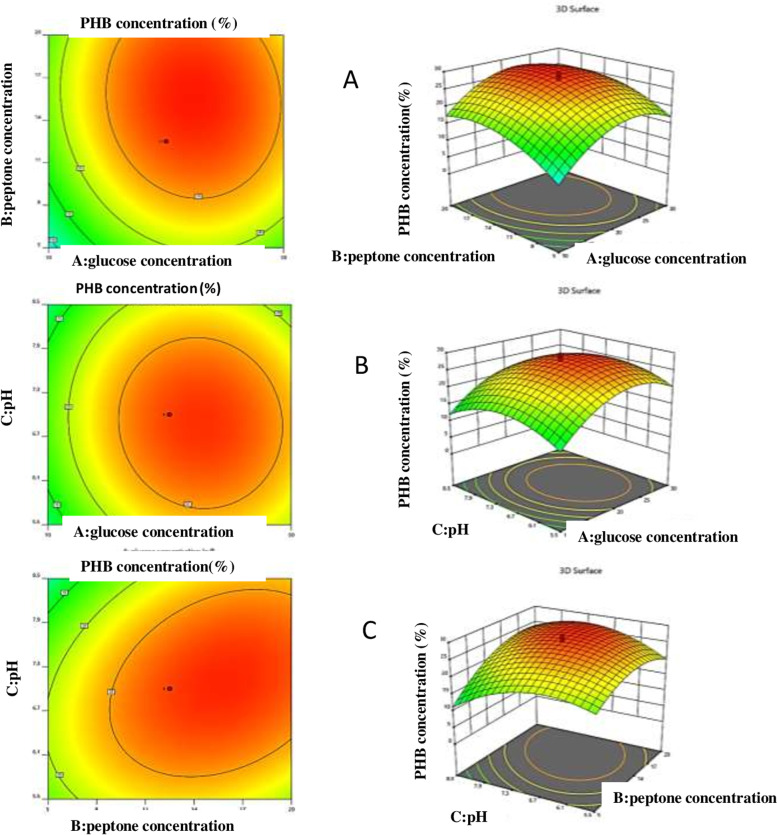
Three-dimensional interactions between of independent factors (**A**) glucose concentration and peptone concentration at standard pH, (**B**) glucose concentration and pH level at standard peptone concentration, (**C**) peptone concentration and pH level at standard glucose concentration

Similarly, response 2 of PHB concentration was studied. The ANOVA for the quadratic model of response 2 (PHB concentration) was tabulated in (Table 3). The 3D plots showed significant influence on PHB concentration by *Bacillus cereus* SH-02 (OM992297) either independently or in the interaction with each other (Fig. 4). Response 2 of PHB concentration (%) was studied and the second-order polynomial equation was given below:

$$\mathrm{Response}\ 2:\mathrm{PHB}\ \mathrm{concentration}\ \left(\mathrm{Y}\right)=+27.65+3.48\ast \mathrm{A}+3.64\ast \mathrm{B}-0.6560\ast \mathrm{C}+0.6687\ast \mathrm{A}\ast \mathrm{B}-0.5562\ast \mathrm{A}\ast \mathrm{C}+2.71\ast \mathrm{B}\ast \mathrm{C}-6.89\ast {\mathrm{A}}^2-4.29\ast {\mathrm{B}}^2-5.19\ast {\mathrm{C}}^2$$

**Table 3:** ANOVA for Quadratic Model (Response 2: PHB concentration)

Source	Sum of Squares	Df	Mean Square	***F***-value	***p***-value
**Model**	1545.29	9	171.70	55.41	< 0.0001^a^
**A-glucose concentration**	165.37	1	165.37	53.37	< 0.0001^a^
**B-peptone concentration**	180.47	1	180.47	58.24	< 0.0001^a^
**C-pH**	5.88	1	5.88	1.90	0.1985 NS
**AB**	3.58	1	3.58	1.15	0.3078 NS
**AC**	2.48	1	2.48	0.7989	0.3924 NS
**BC**	58.59	1	58.59	18.91	0.0014^b^
**A**^**2**^	683.45	1	683.45	220.57	< 0.0001^a^
**B**^**2**^	264.97	1	264.97	85.51	< 0.0001^a^
**C**^**2**^	388.11	1	388.11	125.25	< 0.0001^a^
**Residual**	30.99	10	3.10		
**Lack of Fit**	22.83	5	4.57	2.80	0.1415 NS
**Pure Error**	8.15	5	1.63		
**Cor Total**	1576.27	19			
**Std. Dev.**	1.76				
**Mean**	16.48				
**C.V. %**	10.68				
**PRESS**	194.13				
**R**^**2**^	0.9803				
**Adjusted R**^**2**^	0.9627				
**Predicted R**^**2**^	0.8768				
**Adeq Precision**	21.6548				

^a^, Highly significant; ^b^, Significant; *NS* Not significant

The model F-value of 55.41 implies the model is significant. In this case, A, B, BC, A^2^, B^2^, and C^2^ are found to be significant model terms. The interactive effect of variables, BC was found to be the most significant on response 2 (Fig. 4A–C) compared to other variables that had a negative effect. Glucose concentration and peptone concentration had shown the highest influence on PHB concentration compared to pH (Table 3). The (Lack of Fit) F-value (2.8) is insignificant relative to the pure error. There is 14.15% chance that (lack of Fit) could occur due to noise. The total determination coefficient R^2^ value was 0.9803, indicating an accurate model fit to the experimental data [17]. This also implies that 98% of response variance can be properly explained and that only 2% of the fluctuations arise during the studies. With a coefficient of variation of 10.68%, the adjusted determination coefficient value of 0.9627 revealed that the model was highly significant. The Predicted R^2^ of 0.8768 was reasonably close to the adjusted R^2^ of 0.9627, demonstrating that the model is really important. The ratio of 21.655 suggests that the model can explore the design space (Table 3).

**Fig. 5:**
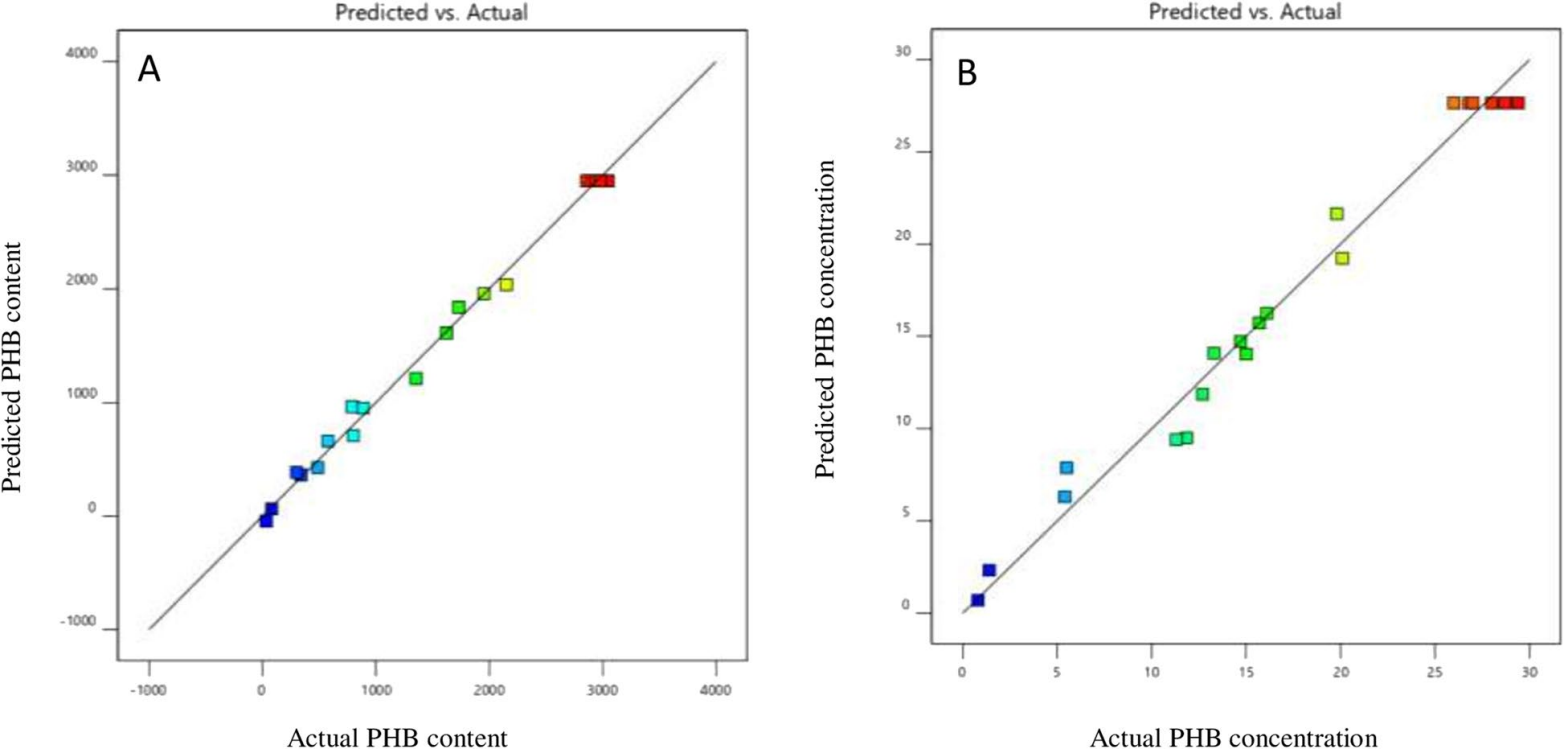
The correlation between predicted and actual value for (PHB content) (**A**) and (PHB concentration) (**B**)

The predicted and actual (experimental) PHB production responses were nearly identical. The expected PHB content and concentration values were derived using regression analysis and compared to experimental data, indicating that the actual response values were consistent with the projected response values. The replies’ predicted versus actual plots were depicted in (Fig. 5A & B). Table 4 showed the suggested solution for the constraint was glucose concentration (22.315 g/L), peptone concentration (15.625 g/L) and pH (7.048) to maximize PHB content (3100.799 mg/L) and concentration (28.799%).

**Table 4:** Constraints, criteria and solution for optimization of PHB production

Name	Goal	Lower limit	Upper limit	Importance	Solution
A:Glucose concentration	In range	10	30	3	22.315
B:Peptone concentration	In range	5	20	3	15.625
C:pH	In range	5.5	8.5	3	7.048
PHB content	Maximize	34	3051	3	3100.799
PHB concentration	Maximize	0.8	29.4	3	28.799

### Polymer analysis

#### FTIR spectroscopy

**Fig. 6:**
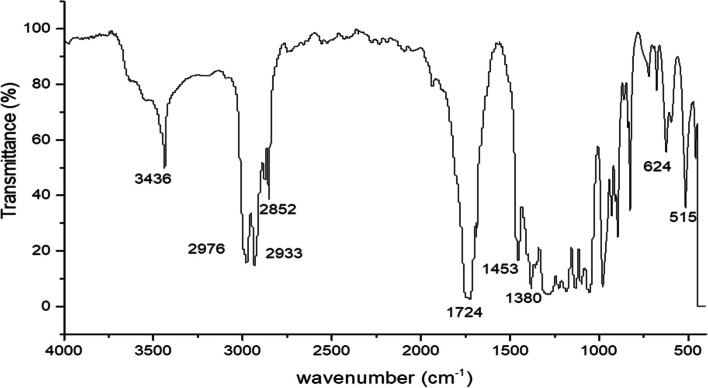
FTIR analysis of PHB extracted from *Bacillus cereus* SH-02 (OM992297)

Polymer extracted from *Bacillus cereus* SH-02 (OM992297) was characterized by FTIR spectroscopy (Fig. 6) to identify the chemical functional group of PHB. A strong band that appeared at 1724 cm^− 1^ is attributed to the ester group’s carbonyl (C=O) stretching. The band at 1453 cm^− 1^ corresponds to the asymmetrical deformation of the C–H bond in CH_2_ groups while the band at 1381 cm^− 1^ corresponds to CH_3_ groups. The terminal OH groups created the band at 3436 cm^− 1^. The bands at 2934 and 2976 cm^− 1^ were assigned to C–H stretching methyl and methylene groups; respectively [18]. The peak at wavenumber 1057.58 cm^− 1^ and 979.88 cm^− 1^ reveal the presence of alkyl halides which confirm the polymer is PHB.

### Mass-spectroscopy

**Fig. 7:**
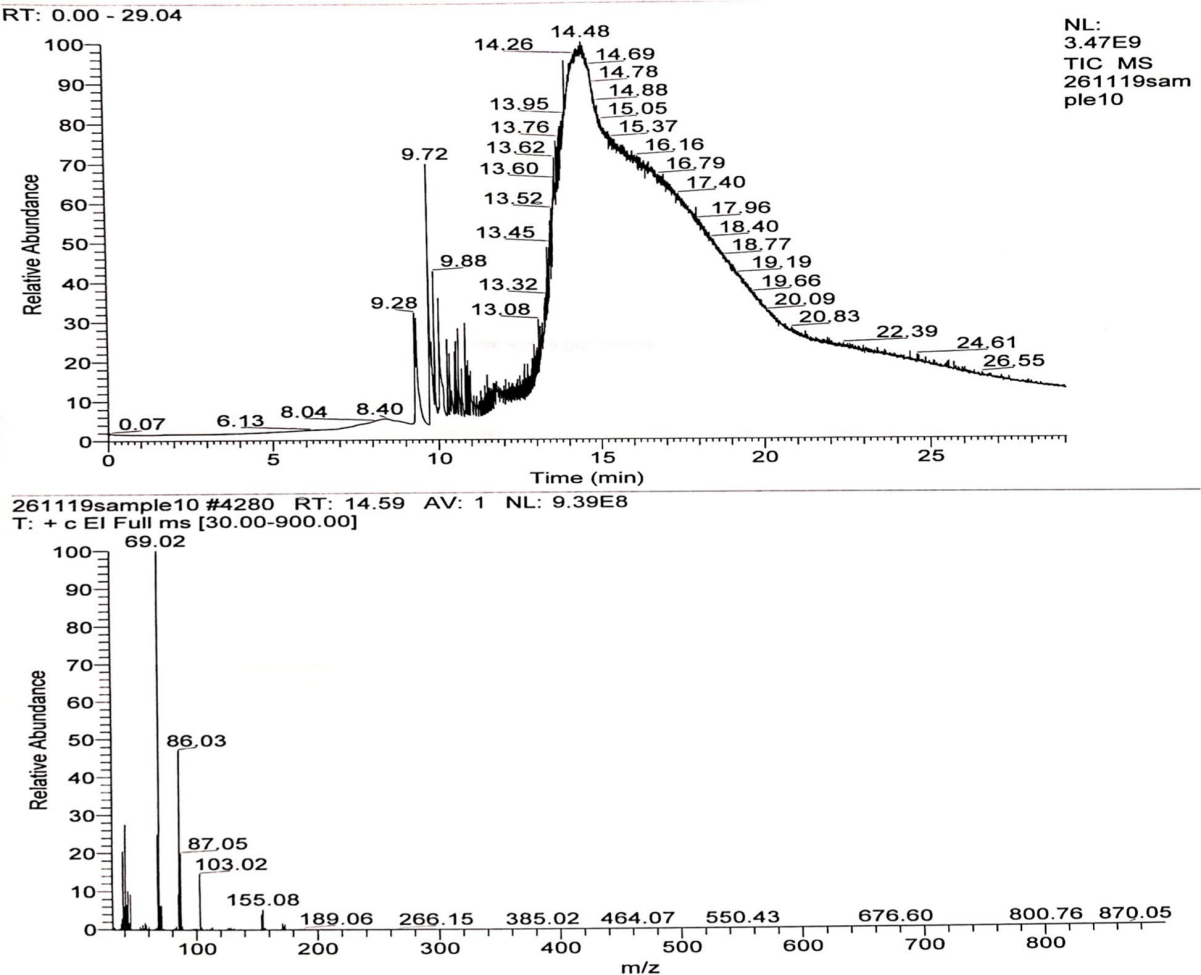
Mass spectra of polyhyroxy butyrate (PHB) extracted from *Bacillus cereus* SH-02 (OM992297)

The relative abundance of an ion is plotted against the *m*/*z* value (Fig. 7). The molecular peak [M]^•+^ corresponds to the compound’s *M r* and the base peak is the most intense one in the spectrum. The peak at m/z 103.03 in the mass spectrum of methyl 3- hydroxybutyric acid represented its hydroxyl end [M^•+^ − 1] [19]. The peak at m/z 87 in the mass spectrum was represented by methyl butyrate CH_3_-CH^+^-CH-COOH [M^•+^ − OH]. The peak at m/z 86 in the mass spectrum represented butanoic acid CH_3_-CH^=^CH-COOH [M^•+^ − H_2_O]. The peak at m/z 69 in the mass spectrum was represented by CH_3_-CH_=_CH-C_=_O [M^•+^ -H_2_O-OH].

### Nuclear magnetic resonance (NMR) spectroscopy

**Fig. 8:**
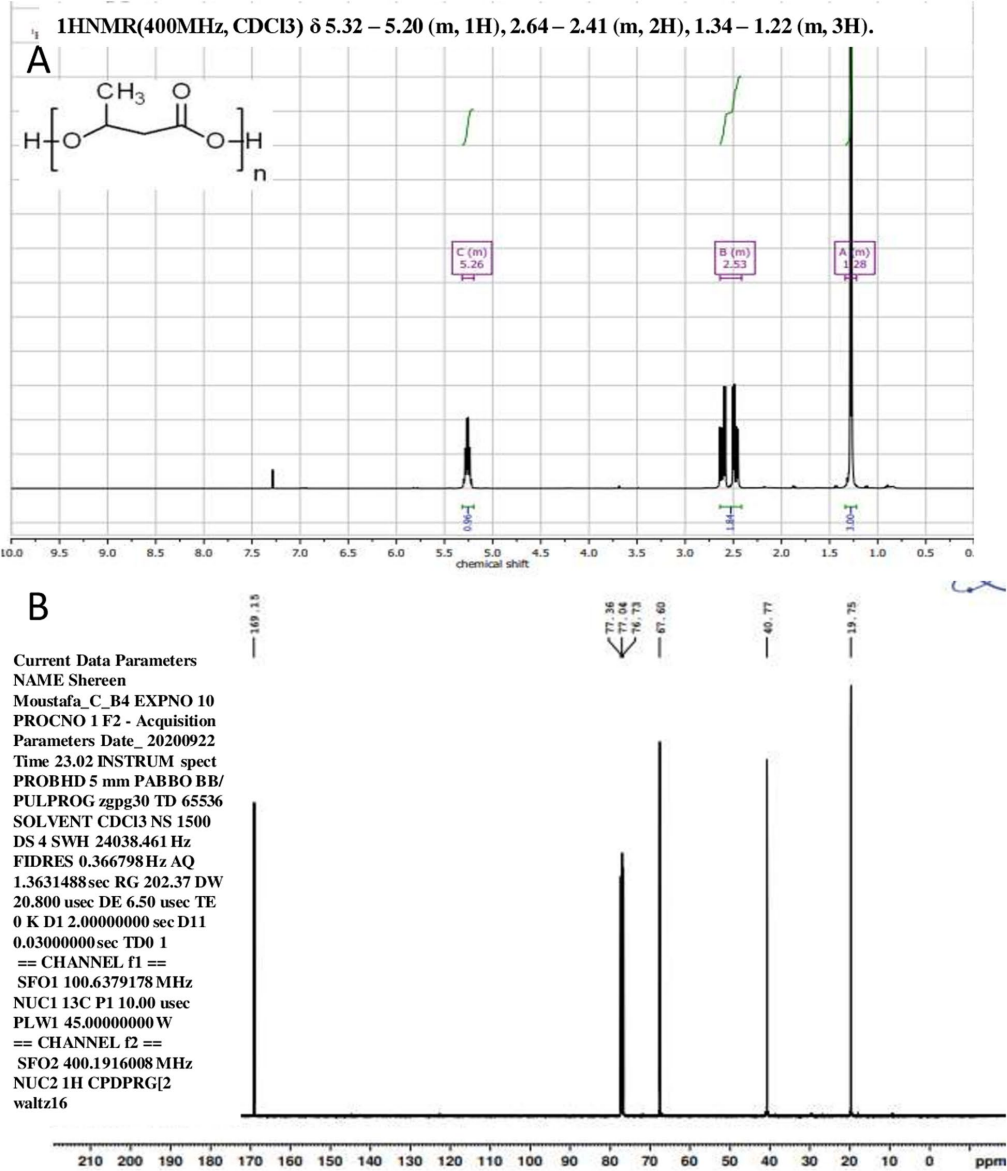
^1^H NMR (**A**) and ^13^C NMR (**B**) Spectra of Polyhydroxybutyrates extracted from *Bacillus cereus* SH-02 (OM992297)

NMR analysis was performed to confirm the biochemical structure of PHB (Fig. 8A&B). ^1^H NMR spectrum of purified PHB showed signals of a chemical shift at δ = 1.22–1.34 ppm as a doublet of the methyl group, and a pair of quadruplets at δ = 2.41–2.64 ppm, which is characteristic of a methylene group (CH_2_) linked to the carbonyl group (Fig. 8A). Multiple signals appear at δ = 5.20–5.32 ppm, characteristic of a methine group (−CH).

The ^13^C NMR spectra of the polymer extracted from *Bacillus cereus* SH-02 (OM992297) exhibited peaks at 169.15, 67.6, 40.77, and 19.75 ppm corresponding to different types of carbon atoms present in PHB (carbonyl, methine, methylene, and methyl resonance).

## Discussion

In this study, PHB-producing bacterium was successfully isolated from the Dirout channel at Assiut governorate. This isolate was identified according to phenotypic characterization and 16srRNA as *Bacillus cereus* SH-02 (OM992297). This study showed that the most PHB content for *Bacillus cereus* strain was achieved after 72 h of incubation time. This result was in agreement with the result obtained by Singh [20], who obtained a maximum PHB of 5.311 g/L after 72 h from *Bacillus* sp. Ahmady-Asbchin [21] achieved the highest amount of biomass (0.221 g/L) and PHB (0.080 g/L) from *Bacillus megaterium* using glucose as a carbon source at 37 °C, 150 rpm speed after 72 h of incubation time. *Bacillus cereus* SH-02 (OM992297) produced the maximum PHB amount at 35 °C. This result correlates with the results of Yasin [22] who obtained the highest PHA production using 2% glucose and 1% nitrogen source at 35 °C and pH 7 from *Bacillus cereus* ARY73. Glucose and peptone were found to be the best carbon and nitrogen source for maximum PHB production. This result was supported by the previous result obtained by Alshehrei [23] from *Bacillus cereus* which has the highest PHB content (1.5 ± 0.03 g/L) using glucose as a carbon source. Also, Mohanrasu [24] achieved a maximum PHB yield of 2.74 g/L from *Bacillus megaterium* using glucose as a carbon source at pH (7). Glucose is an easy-to-use carbon source and encourages bacteria to produce more PHB. According to this study, it can be concluded that simple sugars such as glucose are readily used by bacteria and enhance PHB production. In contrast, complex molecules such as starch are not readily used by significant PHB-producing bacteria [25]. In line with this study, peptone has been reported as a suitable nitrogen source for PHB production by *Erythrobacter aquimaris* [18]*.* This study is supported by the previous study conducted by Narayanan et al. [15] on *Bacillus mycoides* DFC1, which proved that complex organic nutrients are the favourest for PHB production.

RSM analysis was applied to improve PHB production. From the present study, it was obvious that all parameters significantly impact PHB content and concentration. Maximum PHB production was observed in the middle levels of both glucose and peptone concentration, while further increases in concentrations resulted in a gradual decrease in PHB production. This study proved that PHB content was increased by 1.4 fold when using RSM optimized medium in comparison to other classical optimization processes. The results attained were in agreement with Mohd Zain et al. [26] who reported that PHB content improved to 1.25 fold compared to using non-optimized media. Previously, RSM has been used as a powerful tool that improved the PHB production by 2.3 fold from *Rhodococcus* sp. strain BSRT1–1 [16]. The predicted and actual (experimental) PHB production responses were nearly identical which approves that the predicted optimum condition was perfect for the production of PHB from *Bacillus cereus* SH-02 (OM992297). The maximum PHB content and concentration were achieved (3100.7 mg/L and 28.7%) using RSM optimized media which were correlated with the experimental value (3051 mg/l and 28.7%). The suggested solution obtained from this study for the constraint to maximize PHB content and concentration was 22.315 g/L glucose, and 15.625 g/L peptone at pH 7.048. It was agreed with the previous research made by Narayanan [15] on *Bacillus mycoides* DFC1 which has maximum PHB yield (3.32 g/L) at glucose concentration 17.34 g/l, peptone concentration 7.03 g/l, and pH 7.3. Another previous study was done by Das [27] on *Bacillus pumilus* AHSD 04 who confirm that Combined optimized conditions for growth and PHB production were recorded as 37.7 g/L glucose, 4.3 g/L tryptose, and pH of 6.8 which enhancement of PHB production to (5.36 g/L).

The total determination coefficient R^2^ value for response1 and response2 were recorded as 0.9941and 0.9803, respectively. The R^2^ is a measure of the quality of fit, with a value ranging from 0 and 1. The stronger the model and the better it predicts the response, the closer the R^2^ is to unity. Otherwise, lower R^2^ values suggest that the response variables are insufficient to explain the variation. The R^2^ values in this investigation showed that both models could account for more than 98% of the variation in PHB content (Y1) and concentration (Y2). The adjusted R^2^ values for response1 and response2 were 0.9888 and 0.9627 which corrects the R^2^ value for the sample size and number of terms [27].

The characters of purified PHB extracted from *Bacillus cereus* SH-02 (OM992297) are very similar to the previous polymer extracted from *Bacillus drentensis* strain BP17 [28]. FTIR spectroscopy of extracted PHB exhibited various bands 1724 cm^− 1^, 1453 cm^− 1^, 1381 cm^− 1^, and 3436 cm^− 1^ which correspond to (C=O) ester, C–H, CH_3_, OH group. This result was interconnected to the findings of Priyanka [17], who obtained PHB from *Bacillus endophyticus* MTCC 9021. The observed peaks in the mass spectroscopy at m/z 103.03, 87, and 69 represented methyl 3- hydroxybutyric acid, butanoic acid, and CH_3_-CH_=_CH-C_=_O. The fragmentation patterns agreed with the result given by Sabarinathan [5]. Further structural analysis of purified PHB was carried out using ^1^H NMR and ^13^C NMR. ^1^H NMR spectrum showed signals of a chemical shift at δ = 1.22–1.34 ppm, δ = 2.41–2.64 ppm and δ = 5.20–5.32 ppm represented to methyl group, methylene group (CH_2_) and a methine group (−CH) [29]. The ^13^C NMR spectra of the polymer exhibited peaks at 169.15, 67.6, 40.77, and 19.75 ppm corresponding to (carbonyl, methine, methylene, and methyl resonance) [3]. The chemical shift signals obtained from this polymer agree with those obtained by Mostafa et al. [18] from *Erythrobacter aquimaris.*

## Conclusion

In conclusion, this study successfully isolated PHB-producing isolate from the Dirout channel at Assiut Governorate. This bacteria was assessed for physiological and biochemical characterization. The result proved that the isolate has a rod shape with an endospore. It was Gram-positive, aerobic, and motile and had some characteristics such as a positive response toward catalase and oxidase test. This isolate was identified as *Bacillus cereus* SH-02 (OM992297) according to 16 S rRNA analyses. Statistical optimization by one-way ANOVA was used to detect the optimum incubation time, temperature, and the best carbon and nitrogen source for PHB production. This study proved that incubation time and temperature have a significant effect on PHB production. The production factors (carbon and nitrogen CONC. and pH) were optimized using RSM with rotatable CCD. Under optimum conditions from RSM analysis, maximum PHB content and concentration can reach (3100.799 mg/L and 28.799%) which was in close agreement with the actual value (3051 mg/l and 28.7%). Confirmation of the extracted biopolymer as PHB was done by FTIR, NMR, and Mass spectrometry. FT-IR analysis exhibited a specific peak at 1724 cm^− 1^ that corresponds to the function group of PHB while NMR analysis has different peaks at 169.15, 67.6, 40.77, and 19.75 ppm that were corresponding to carbonyl, methine, methylene, and methyl resonance. Mass spectroscopy exhibited molecular weight for methyl 3- hydroxybutyric acid.

## Materials and methods

### Isolation and identification of PHB-producing organisms

PHB-producing bacteria were isolated from Dierout channel at Assiut Governorate [30]. This isolate was identified according to its morphological and physiological characterization and 16S rRNA.

### Phenotypically identification

Preliminary identification was performed according to methods described in Bergey’s Manual [31]. The isolate was identified morphologically using a light microscope to observe microbiological indicators such as Gram reaction, motility, and spore formation. Biochemical characterization of the isolate was performed using biochemical assays including carbohydrate fermentation, hydrogen sulfide production, catalase assay, indole production assay, citrate utilization test, urease, starch hydrolysis, gelatin, and casein hydrolysis. The oxidase and deoxidization activities of nitrate were also examined [32].

### Phylogenetically identification

The PHB-producing isolate was identified according to the 16S rRNA sequence. DNA was extracted according to Hesham [33]. PCR amplification was carried out using forward primer (27F): 5′AGAGTTTGATCCTGGCTAG 3′ and reverse primer (1492R): 5′GGCTA CCTTGTTACG ACTT 3′. The GeneBank database in the BLAST program of the National Center for Biotechnology Information was used to compare the sequence of the 16S rRNA gene, which was deposited in GenBank. The phylogenetic tree was built with MEGA version 4.0 (Auckland, New Zealand) using a neighbor-joining approach to establish the taxonomic position of the isolates, plus the Jukes-Cantor distance estimation method with bootstrap analyses for 100 replicates was performed [34].

### Extraction of PHB

PHB-producing strain was inoculated in production media supplemented with an excess amount of carbon source (g/L); 20 glucose, 0.2 MgSO_4_, 0.1 NaCl, 0.5 KH_2_PO_4_, 4.0 peptone, and 2.5 yeast extract [35]. The culture was incubated under shaking (150 rpm) at 30 °C. Then, the polymer was extracted from the bacterial cell by the chloroform/methanol method [30]. After incubation time the culture was centrifuged (MPW-260 Refrigerated Laboratory Centrifuge, Germany) at 5000 rpm for 15 min. The Cell pellet was washed twice with distilled water and dried in a hot air oven (DZF-6020 Laboratory vacuum dry oven) overnight at 60 °C and the dry cell weight was determined by the gram. Dried bacterial cells were treated with sodium hypochlorite (4%) at 50 °C for 2 h. The mixture was centrifuged again at 5000 rpm for 10 min. The pellet was washed with water, acetone, and methanol; respectively. The pellet was dissolved in chloroform at 40 °C for 2 h, then filtrate using (Whatman no. 1, WHA1001045, Buckinghamshire, UK) filter paper to remove nondissolved matter. Chloroform was evaporated at room temperature and obtained pure PHB which was stored for further analysis PHB was weighed in grams and related to the cell dry weight. The PHB concentration was expressed as % of PHB from cell dry weight.

### Effect of media components and process parameters

To achieve higher PHB production, various parameters (incubation time, temperature, and different carbon and nitrogen source) were studied using the one factor at a time method.

### Effect of incubation time on PHB production

To obtain the maximum PHB yield, a conical flask containing 200 ml of production media was inoculated with 5% of the selected strain and incubated at 30 °C, pH (7), and shook at 150 rpm. PHB content of 50 ml bacterial culture was detected at different incubation times (24, 48, 72, and 96 h).

### Effect of temperature on PHB production

For analyzing the effect of temperature on PHB production, 50 ml of sterile production medium was prepared in a different conical flask and inoculated with 5% inoculums. Each flask was incubated for 72 h at different temperatures 25 °C, 35 °C, and 45 °C then, PHB content was determined.

### Effect of different carbon sources on PHB production

To detect the optimum carbon source for PHB production, 50 ml of production media were prepared in different conical flasks supplemented with 2% of various carbon sources (glucose, fructose, sucrose, lactose, maltose, and starch). PHB content for each conical flask was determined after incubation at 35 °C for 72 h.

### Effect of different nitrogen sources on PHB production

To detect the best nitrogen sources for maximum PHB production, 50 ml of production media were prepared in different conical flasks supplemented with 0.4% of different nitrogen sources (peptone, yeast extract, urea, ammonium sulfate, and ammonium chloride) and incubated for 72 h at 35 °C, then, PHB content was determined.

### Optimization of PHB production using response surface methodology (RSM)

Response surface methodology (RSM) (Stat-Ease, Inc. Design-Expert software, trial version, 12) was used to optimize the screened variables for enhanced PHB production. According to the preliminary experiments regarding the effect of the parameters on PHB production, the chosen variables were glucose concentration, peptone concentration, and pH. The prediction optimum value of factors that affected PHB production is shown in supplementary data (Fig. S1). The experimental combination of selected factors was investigated at −α (− 1.68), high (+ 1), medium (0), low (− 1), and + α (+ 1.68) levels. The design was used to find the optimum condition of the most significant bioprocess variables: glucose concentration (10, 20 and 30 g/L), peptone concentration (5, 12.5 and 20 g/L), and pH level (5.5, 7 and 8.5). PHB content and concentration were studied as the experimental response using analysis of variance (ANOVA) to determine the interactive effects of the three variables. The experiment design for the tested variables is depicted in (Tables 5 and 6). The response surface model graphs were used to identify the effects of linear, quadratic and interactive terms of the independent variables on the chosen dependent variables [15]. The F-value is also checked to determine the significance of all the fitted equations at a 5% level of importance [36].

**Table 5:** Test variables and levels of central composite design (CCD) to optimize glucose concentration, peptone concentration and pH level for the polyhydroxyalkanoate production by *Bacillus cereus* SH-02

			Coded levels				
ariable	Symbol	Unit	-α (−1.68)	Low (−1)	Middle (0)	High (1)	+α (+ 1.68)
Glucose concentration	A	g/l	3.2	10	20	30	36.8
Peptone concentration	B	g/l	−0.11	5	12.5	20	25.11
PH levels	C	–	4.5	5.5	7	8.5	9.5

**Table 6:** Combination of the experiment based on CCD for optimization of glucose concentration, peptone concentration, and pH level in the production of PHB by *Bacillus cereus* SH-02

Run no.	Factors			Response	
	A:glucose concentration(g/L)	B:peptone concentration(g/L)	C:pH	PHB content(mg/L)	PHB concentration(%)
1	20	12.5	7	2880	26
2	30	5	8.5	300	5.5
3	20	12.5	7	2980	27
4	3.18207	12.5	7	82	1.4
5	20	12.5	7	2940	26.8
6	20	12.5	7	3051	28.7
7	30	5	5.5	1356	15.7
8	10	5	8.5	34	0.8
9	10	20	5.5	488	11.85
10	30	20	8.5	1954	20.1
11	20	−0.113446	7	578	11.3
12	20	12.5	4.47731	890	13.3
13	20	25.1134	7	2152	19.8
14	10	5	5.5	340	5.4
15	30	20	5.5	1732	16.1
16	36.8179	12.5	7	1624	15
17	20	12.5	7	2860	28
18	20	12.5	7	2980	29.4
19	10	20	8.5	792	14.7
20	20	12.5	9.52269	802	12.7

The data in (Table 6) depicts a significant variation of PHB content from 34 to 3051 mg/L and PHB concentration from 0.8 to 29.4%. The PHB content and concentration were further subjected to regression analysis and analyzed as the experimental response using analysis of variance (ANOVA) to determine the interactive effects of the three variables. A mathematical model generated by statistical software was used to determine the optimum conditions of selected variables, which were then validated in the actual experiment. The percentage of deviation between suggested optimal points and actual experimental results was investigated.

### Polymer analysis

PHB produced from *Bacillus cereus* SH-02 was verified and characterized by Fourier Transform Infrared Spectroscopy (FTIR), Mass spectroscopy, and Nuclear Magnetic Resonance (NMR) Spectroscopy.

### Fourier transform infrared spectroscopy (FTIR)

The functional group for PHB was determined by FTIR spectroscopy using Nicollet 6700 FTIR spectrophotometer (Assiut University, Egypt, Thermo Fisher Scientific, 168 Third Avenue, Waltham, MA 02451, USA). Two mg of PHB was mixed with 20 mg of Potassium Bromide (KBr) to make a transparent pellet and ground in a Motor then, Pestle used a hydraulic pressure instrument. IR rays were passed through it at a range of 4000–400 cm-^1^ [24].

### Mass spectroscopy

Mass analysis was performed to determine the monomer composition of the extracted polymer. The polymer was analyzed using a mass spectrometric (Varian, CP–3800 GC and Saturn 2200 MS) at the Analytical Chemistry Unit, Faculty of Science, Assiut University, Egypt. Mass spectrometry was used to convert the molecules into charged fragments called ions, separated by their masses. Mass spectrometry records the ratio of mass to charge (*m*/*z*) on the horizontal axis and the abundance of ions on the vertical axis. Ions of different mass move along a different path before they reach a detector that records the intensity and masses of the ions hitting it.

### Nuclear magnetic resonance (NMR) spectroscopy

To determine the chemical structure for monomer composition of PHB, the sample was subjected to nuclear magnetic resonance (NMR) using Bruker high-Performance Digital FT-NMR Spectrometer Avance 400 MHz proton frequency, at the NMR Unit, Faculty of Pharmacy (Cairo University). The sample was prepared by dissolving 25 mg of extracted PHB in 1 ml of deuterated chloroform (CDCl_3_). Then, it was transferred to a 5 mm diameter NMR tube and subjected to the 400 MHz ^1^H NMR and 300 MHz ^13^C NMR analysis. The proton (^1^H) NMR analysis was carried out using a Bruker Advance 400 NMR, while ^13^C NMR was measured using a Bruker Advance 300 (Bruker Corporation, Tucson, AZ). Tetramethyl saline (TMS) was used as an external reference. Chemical shifts are reported in ppm and coupling constants are reported in Hz. The spectrometer is equipped with broadband direct-detection (BBO) sensors. All NMR measurements were obtained at 298 K (25 °C). Data are analyzed using Topspin 3.1 software (Bruker Biosoin, Rheinstetten, Germany). The following conditions were used to record the ^1^H NMR and ^13^C-NMR spectra: a 30 °C pulse experiment; acquisition time of 4.1 seconds; relaxation delay of 1.0 sec; sweeping width of 15.1 ppm (8012 Hz); Data points 65,536 and dummy scan 2. Data were processed using 0.1 Hz line expansion [26].

## Supplementary Information

**Additional file 1.**

## Data Availability

All data generated or analysed during this study are included in this published article. The nucleotide sequence of 16S rRNA gene sequences of isolated strain reported in this study has been deposited in the GenBank (https://www.ncbi.nlm.nih.gov/genbank/) under the name *Bacillus cereus* and the accession number OM992297.
