# Supplementary material for: Production and optimization of bioplastic (Polyhydroxybutyrate) from Bacillus cereus strain SH-02 using response surface methodology
Source: BMC Microbiol. 2022 Jul 22;22:183. doi: 10.1186/s12866-022-02593-z (PMC9306189; doi:10.1186/s12866-022-02593-z)
Supplement: Supplementary file 1 — Additional file 1. [file 12866_2022_2593_MOESM1_ESM.docx]

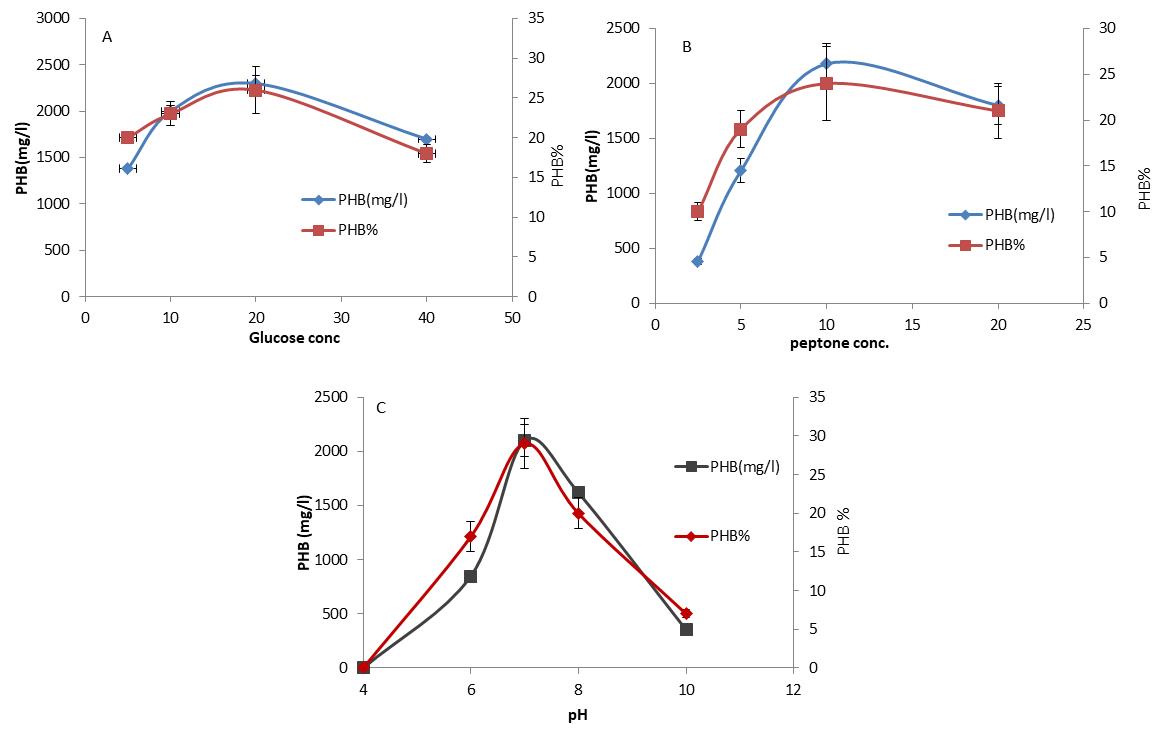


Figure s1: Effect of glucose conc. (A), peptone conc. (B) and pH (C) on polyhydroxy butyrate (PHB) production by *Bacillus cereus* SH-02 (OM992297).
